# Clinical outcomes and periodontal conditions of dental implants placed in free fibula flaps (FFF): a retrospective study with a mean follow-up of 6 years

**DOI:** 10.1007/s00784-023-05364-w

**Published:** 2023-11-02

**Authors:** Giorgio Lombardo, Antonio D’Agostino, Pier Francesco Nocini, Annarita Signoriello, Alessandro Zangani, Alessia Pardo, Fabio Lonardi, Lorenzo Trevisiol

**Affiliations:** Dentistry and Maxillo-Facial Surgery Unit, Department of Surgery, Dentistry, Paediatrics and Gynaecology (DIPSCOMI), University of Verona, Piazzale L.A. Scuro 10, 37134 Verona, Italy

**Keywords:** Dental implants, Oral hygiene, Prosthetic, Survival, Free fibula flap

## Abstract

**Objectives:**

Up-to-date literature regarding long-term success of implant rehabilitations after microvascular reconstructions with free fibula flap (FFF) is still very scarce. This study aimed to evaluate clinical outcomes, especially related to oral hygiene conditions, of patients rehabilitated with this technique.

**Materials and methods:**

A total of 25 patients who underwent maxillofacial reconstructive surgery with FFF were retrospectively evaluated for soft tissues conditions, oral hygiene habits, and implant survival and success, assessed with a mean follow-up of 6 (range 2–15) years after loading.

**Results:**

Fourteen patients received full-arch fixed prostheses and 11 removable bar-supported overdentures. At the follow-up evaluation, 52% of prostheses did not allow proper accessibility for oral hygiene. Overall prosthetic survival was 100%, and implant survival and success were respectively 93.6% and 72%. Prevalence of peri-implantitis was 29% at implant level and that at patient level 96%.

**Conclusions:**

Six-year clinical outcomes of this study reveal that poor oral hygiene practices and compliance by patients who underwent maxillofacial reconstruction with FFF are significantly associated with peri-implant disease.

**Clinical relevance:**

Findings of the present study underline the need by clinicians for a careful assessment, in reference to a specific implant therapy, of patient’s prosthetic accessibility for oral hygiene procedures.

## Introduction

Patients characterized by maxillofacial defects, resulting from head and neck cancer, facial trauma, severe atrophies, or congenital diseases, always require a challenging treatment planning for oral rehabilitations: complications arising from these severe defects include facial deformity and compromised main oral functions, such as speech, mastication and swallowing [1]. Even if dental implants may significantly contribute to restore an adequate overall functionality, fixtures of standard dimensions cannot be used in most of the abovementioned cases, for lack of sufficient bone levels [2]. Several options have been developed over the years to correct deficient edentulous ridges, including short or reduced diameter dental implants [3, 4]; tilted dental implants [5]; zygoma implants [6]; bone splitting/expansion of narrow ridges [7]; alveolar distraction osteogenesis [8]; guided bone regeneration [9]; and Le Fort I osteotomy with inter-positional bone grafts [2, 10] (in cases of large sagittal discrepancy between the jaws and when implant inclination is too unfavorable). In this proposal, one of the most frequently employed procedures is represented by the reconstruction of alveolar defects with autogenous bone grafts, harvested from intraoral or extraoral sites: this modality of treatment was found to be a reliable means to correct both moderate and severe alveolar bone deficiencies of partially or totally edentulous patients [11, 12]. Nevertheless, the ability of these grafts to maintain the original bone volume is limited by the size of segmented defect continuity, and their survival mostly depends on revascularization from the recipient site and amount of soft tissues available to achieve sufficient graft coverage [13].

In case of patients affected by extensive craniofacial bony defects, whose soft tissues are inadequate, free fibula flap (FFF) [14] was proposed for the reconstruction of maxillary and mandible continuous defects. Despite there being no absolute consensus on when free tissue transferred should be used over a non-vascularized bone graft, current literature suggests the treatment of any segmental defects ≥ 6 cm generally with a revascularized graft, like a FFF [15, 16]. Advantages achieved by using this flap include a sufficient length for the bony segment harvested, a good vascularization, a long vascular pedicle, and proper volumes for implant placement [17]. FFF thus provides strong bi-corticalism to allow implant positioning [18, 19] and more comfortable implant-supported prosthesis rehabilitation at the same time [20].

Nonetheless, transplanted fibula tissue requires considerable quantity of soft tissue (muscle cuffs and/or vascular pedicles): as the skin of the FFF is sometimes very thick and exhibits considerable mobility, such suboptimal peri-implant tissue often promotes inflammation, hypertrophy, pain, bleeding, and finally development of peri-implantitis [21]. Several authors largely highlight that peri-implantitis is directly related to inability to perform proper oral hygiene procedures [22]. Plus, patients who underwent reconstructive surgery suffer from limitations in oral opening because of oral vestibule contracture, reporting difficulty in accessing the abutments for effective home-care oral hygiene procedures [23]. As many studies demonstrated that higher plaque levels [24, 25] and lack of accessibility for cleaning at implant level [21] are both significantly associated with peri-implantitis, oral hygiene performed by patients therefore represents a key component in preventing peri-implant diseases [26, 27].

In light of these considerations, the long-term success of implant rehabilitations after microvascular reconstructions with FFF implies strict personal and professional oral hygiene protocols. To the best of authors’ knowledge, it seems that the efficacy of specific oral hygiene methods and their impact on peri-implant outcomes in this population of patients are not yet well established. This retrospective study aimed to investigate clinical outcomes for patients who underwent maxillofacial reconstruction with FFF, specifically evaluating the importance of oral hygiene habits and instructions.

## Materials and methods

### Study design

The present study was designed as a retrospective clinical study, in compliance with the principles of the Declaration of Helsinki on medical protocol and ethics and good clinical practice guidelines for research on human beings. Ethical approval was obtained from University of Verona Institutional Review Board (protocol code 927CESC, 11/07/16). The nature and aim of the study, together with the anonymity in the scientific use of data, were clearly explained in a written, informative consent form, which was signed by every patient. Patients included in the study underwent mandibular or maxillary reconstruction involving the use of FFF followed by the insertion of dental implants. Inclusion criteria were as follows: patients who had dental implantation and implant-supported rehabilitation after jaw reconstruction with FFF, and documentation of at least two subsequent appointments (one check-up soon after prosthetic rehabilitation, and one clinical and radiographic follow-up examination). A retrospective evaluation was conducted at the Unit of Dentistry and Maxillofacial Surgery of the University of Verona (Italy) on the available medical records possible to find for patients previously involved in the abovementioned maxillofacial reconstructive surgery: patients finally included in the database were those surgically treated with the FFF technique, which was employed between 01/01/1993 and 31/12/2007, satisfying the reported inclusion criteria. The retrospective evaluation of patients’ records consisted in the collection of the following data: patients’ demographics; cause of jaw atrophy (indications for surgery); defect’s location; implant type, number, and location; type of prosthetic rehabilitation provided; panoramic radiographs; soft tissues conditions; oral hygiene habits.

### Surgical protocol for FFF

As previously mentioned, patients underwent reconstructive surgery with FFF in case of extreme atrophies, as defined by the Cawood-Howell classification [2]. Preliminary evaluations consisted in standard clinical examination, panoramic radiograph, computerized tomographic scans, study models, blood tests, angiography of the lower leg, and electrocardiography. All patients underwent pre-operative anaesthesia and cardiac consultations; surgery was then performed under general anaesthesia.

The recipient sites were prepared with a crestal incision from the left to the right retromandibular space in the mandible, and the right to left tuberosities in the maxilla, and the mandibular/maxillary bodies were fully exposed. In all cases, every attempt was made to spare the condyle and temporomandibular joint capsule and the mental foramina in the mandibular arch, and to preserve the greater palatine arteries on the palatal surface. The facial vessels were exposed in the neck via a 3-cm submandibular incision, taking care to not damage the cervical branch of the facial nerve [19]. The fibula flap was harvested using the lateral approach to the anterior compartment of the leg, as described by O’Brien and Morrison (1987) [28]. The flap was modelled according to the anatomy of the recipient sites; osteotomies were performed with a piezoelectrical device or with a reciprocating saw; titanium plates and screws were used for the osteosynthesis of the flap to the recipient sites. The vascular bundle of the fibula was placed along the vestibular side of the new mandibular arch or along the palatal aspect of the new maxillary arch. The pedicle was connected to the facial vessels, and the muscle cuff of the fibular flap was left partially exposed in the mouth to allow free granulation and mucosal colonization to take place [19]. Loose interrupted sutures secured the vestibular and lingual muco-periosteal flaps to the muscle cuff at the maxillary and mandibular levels. The muscle cuff allowed direct monitoring of the flap vitality in the first post-operative days.

### Implant placement and prosthetic protocol

None of the patients underwent implant placement at the time of FFF surgery. Plus, at the time of implant placement, osteosynthesis plates and screws were removed only if they interfered with the desired implant position. All dental implants were screw-shaped, 10, 11.5, and 15 mm in length, and 4.0 and 5.0 mm in diameter. Implant placement followed standard procedures [29] and sutures were removed 14 days postoperatively. All patients received oral postoperative antibiotic therapy (amoxicillin plus clavulanate, 1 g every 8 h), for 10 days postoperatively.

**Fig. 1:**
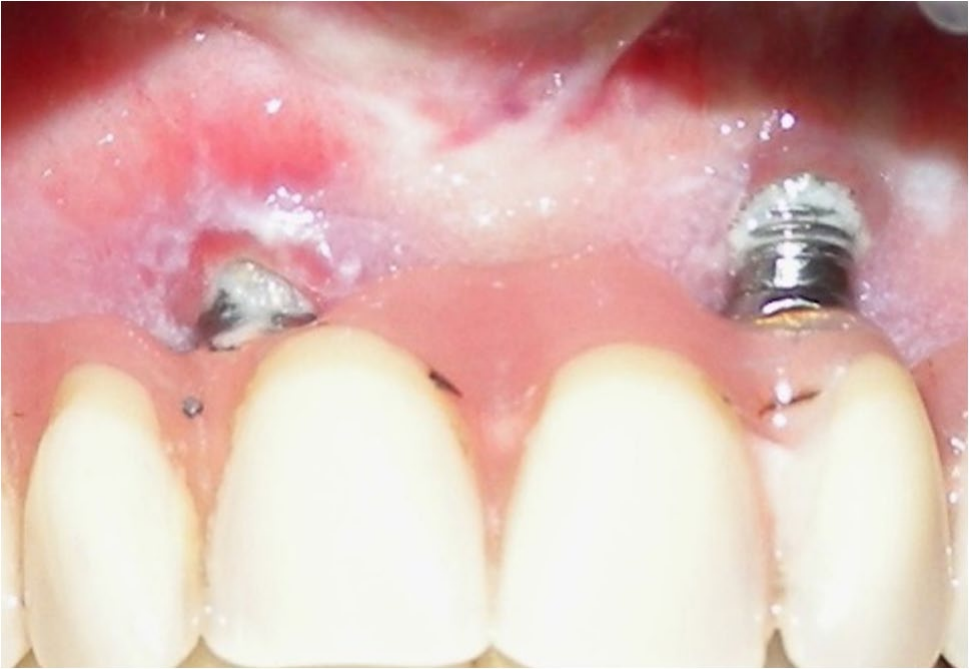
Soft tissues assessment of visible plaque index (VPI)

**Fig. 2:**
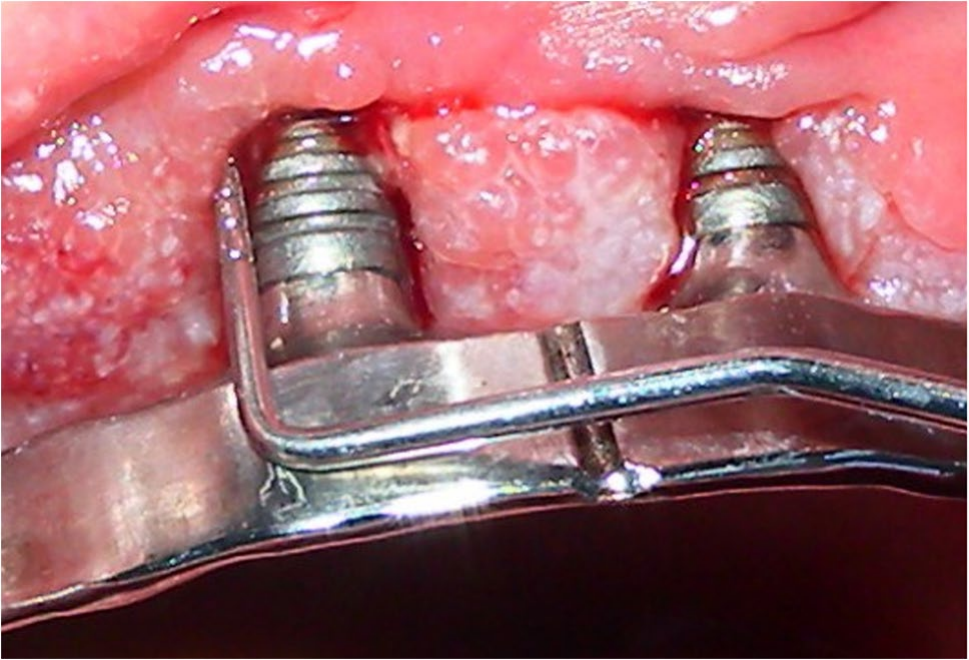
Soft tissues assessment of bleeding on probing (BoP)

**Fig. 3:**
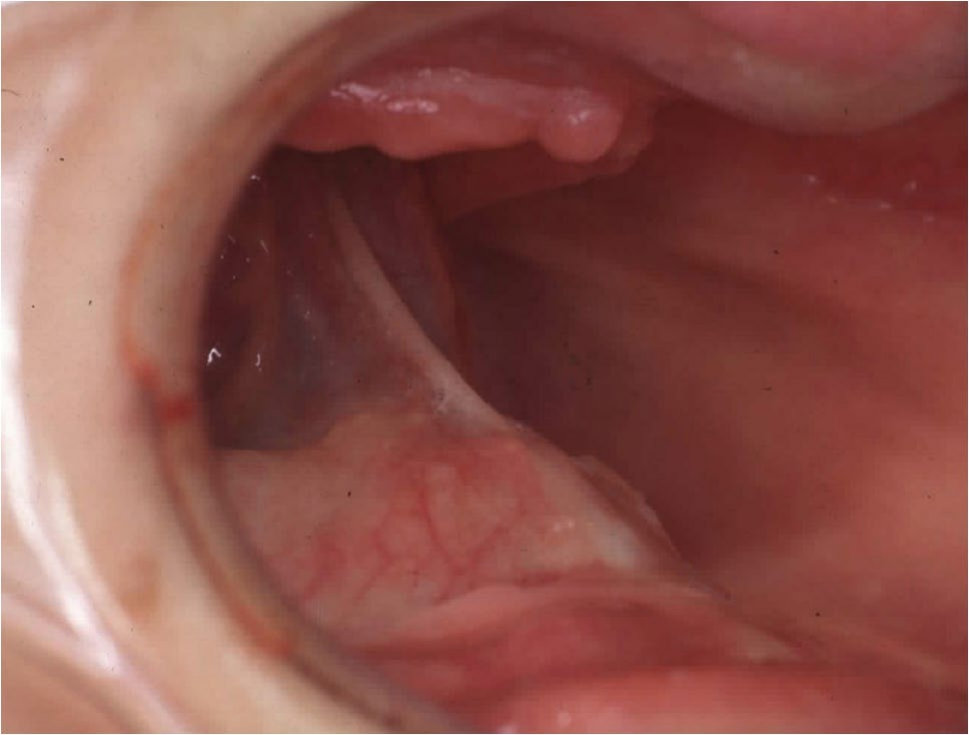
Full-arch prosthetic rehabilitation of a 50-year-old female patient with class VI extreme atrophy. Clinical photographs at pre-surgical time: intraoral view. See extremely resorbed jaws and pseudo-prognathism due to maxillary atrophy

**Fig. 4:**
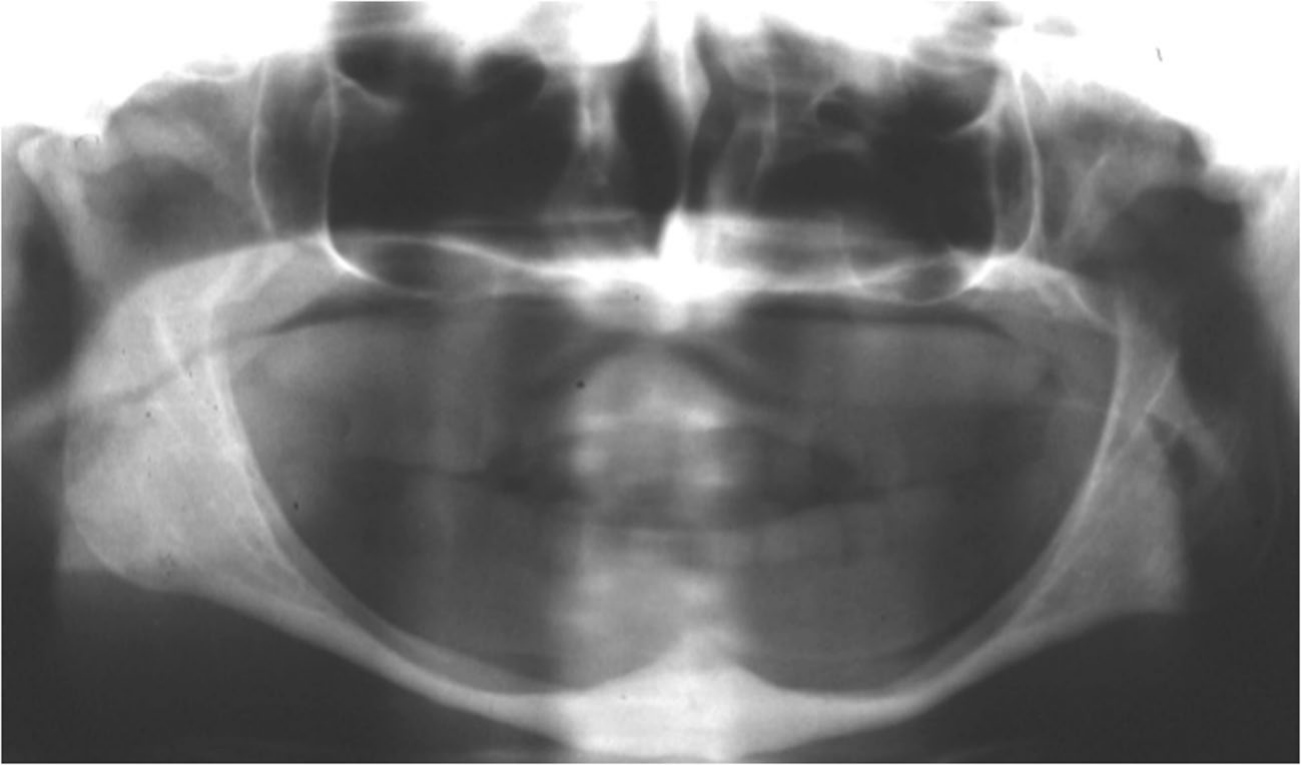
Full-arch prosthetic rehabilitation of a 50-year-old female patient with class VI extreme atrophy. Clinical photographs at pre-surgical time: X-ray panoramic radiograph. See extremely resorbed jaws and pseudo-prognathism due to maxillary atrophy

**Fig. 5:**
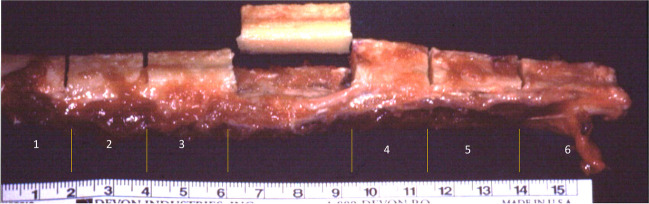
C-Shaped free fibula flap (FFF): harvesting, segmentation, and removal of central segment

**Fig. 6:**
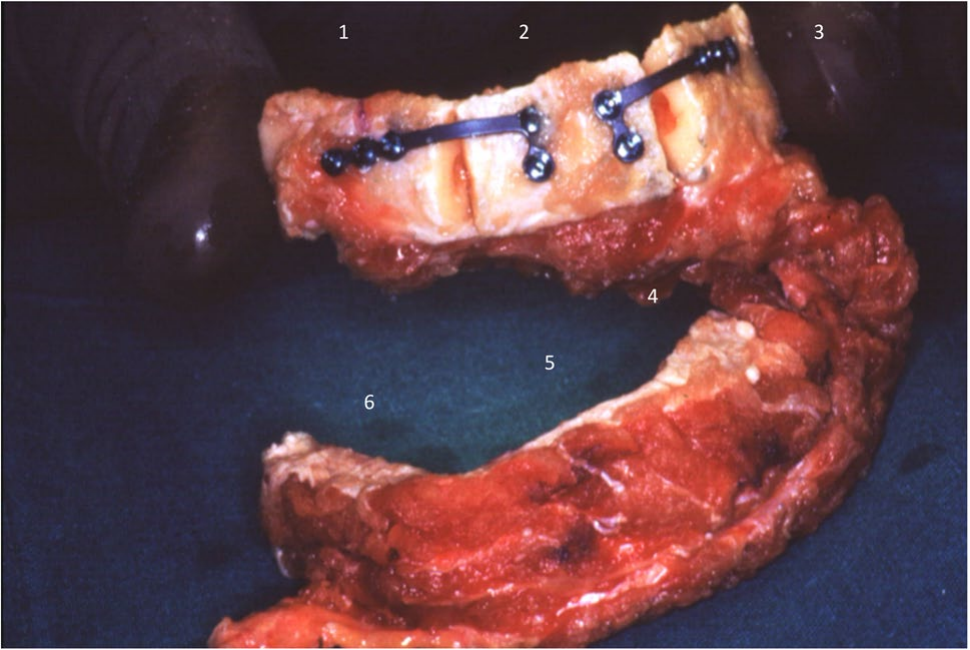
C-Shaped free fibula flap (FFF): shaping of free fibular flap prior to insetting

**Fig. 7:**
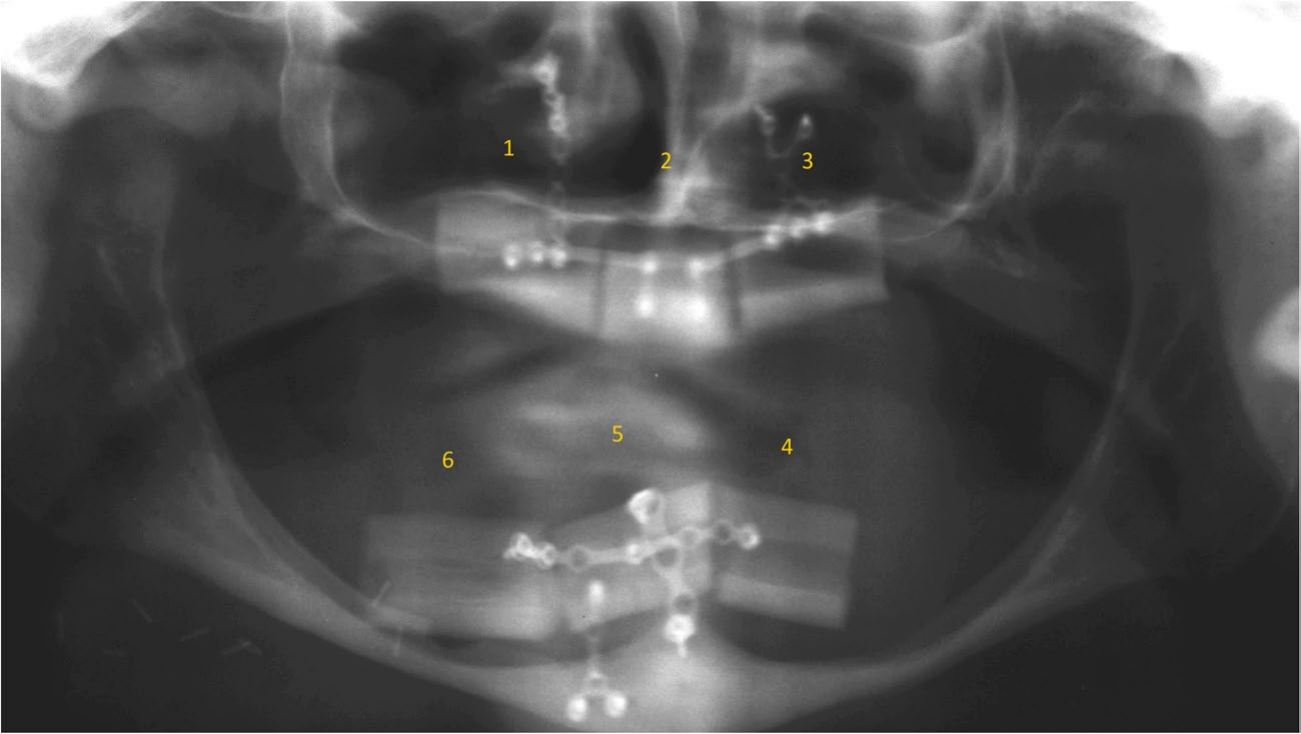
C-Shaped free fibula flap (FFF): X-ray panoramic radiograph taken after FFF placement

**Fig. 8:**
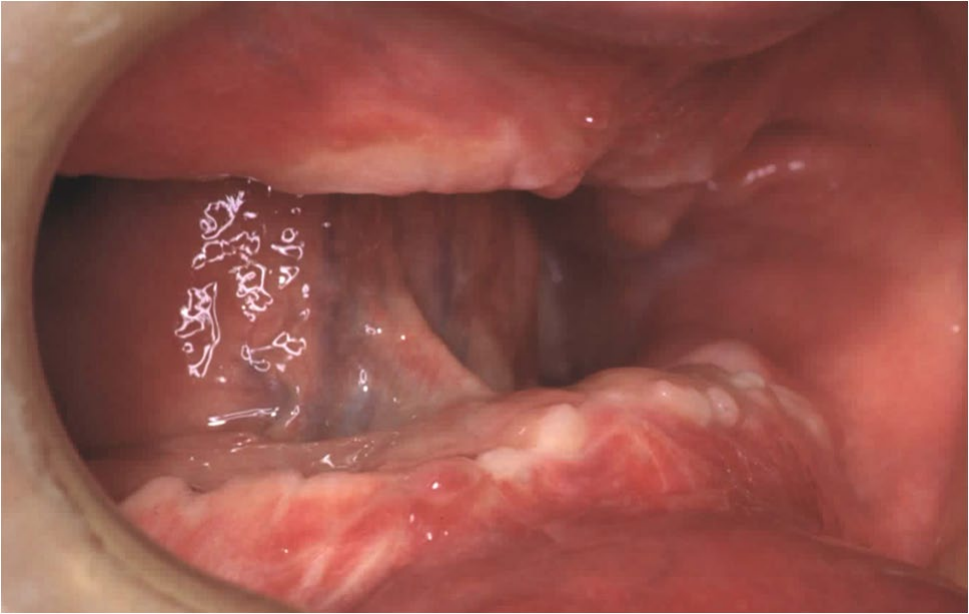
Intraoral view 2 months after surgery; see proper soft tissues healing

**Fig. 9:**
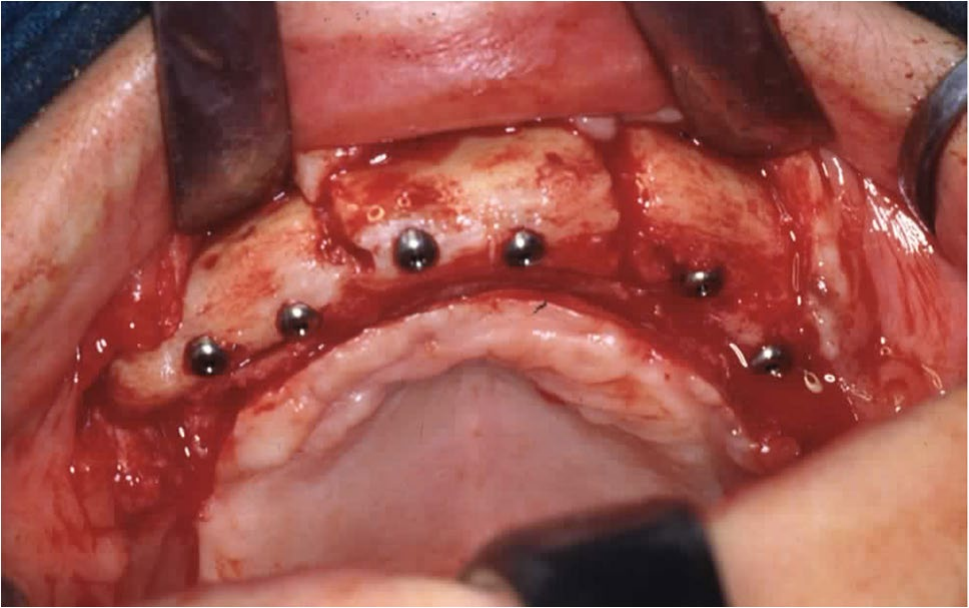
Osteosynthesis plates and screws removal and concomitant surgical flap dissection with implant placement: intraoral view of the upper arch

**Fig. 10:**
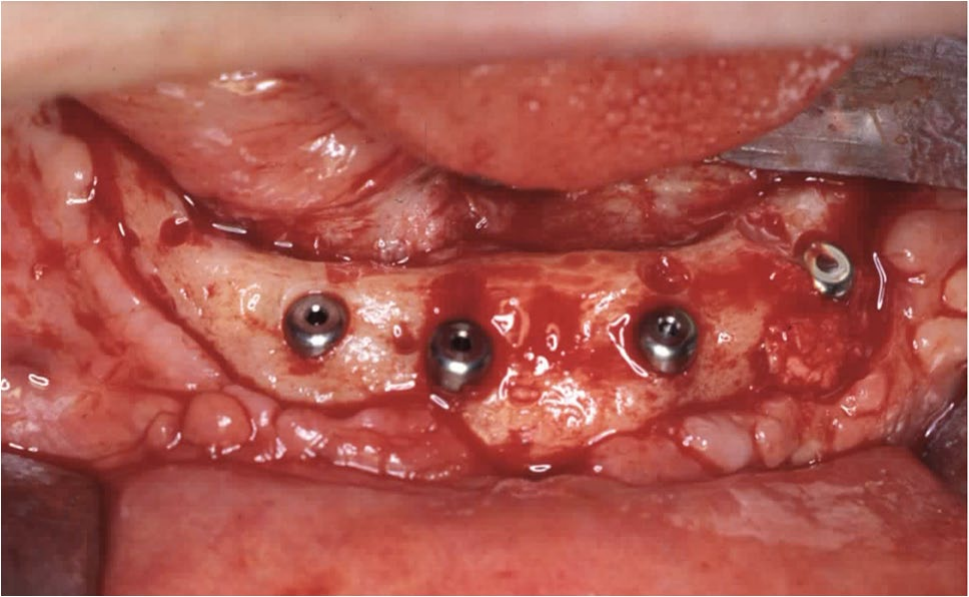
Osteosynthesis plates and screws removal and concomitant surgical flap dissection with implant placement: intraoral view of the mandible

**Fig. 11:**
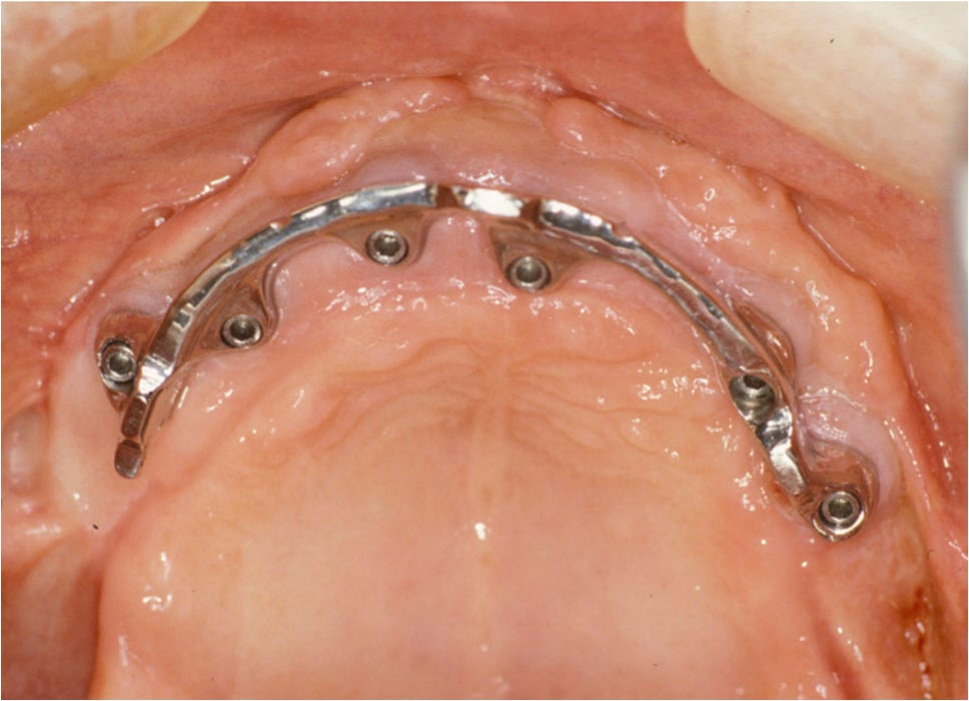
Prosthetic rehabilitation with overdenture 7 months after implant placement: intraoral view of the upper arch; see soft tissues healing after skin graft harvested from the antero-lateral thigh

**Fig. 12:**
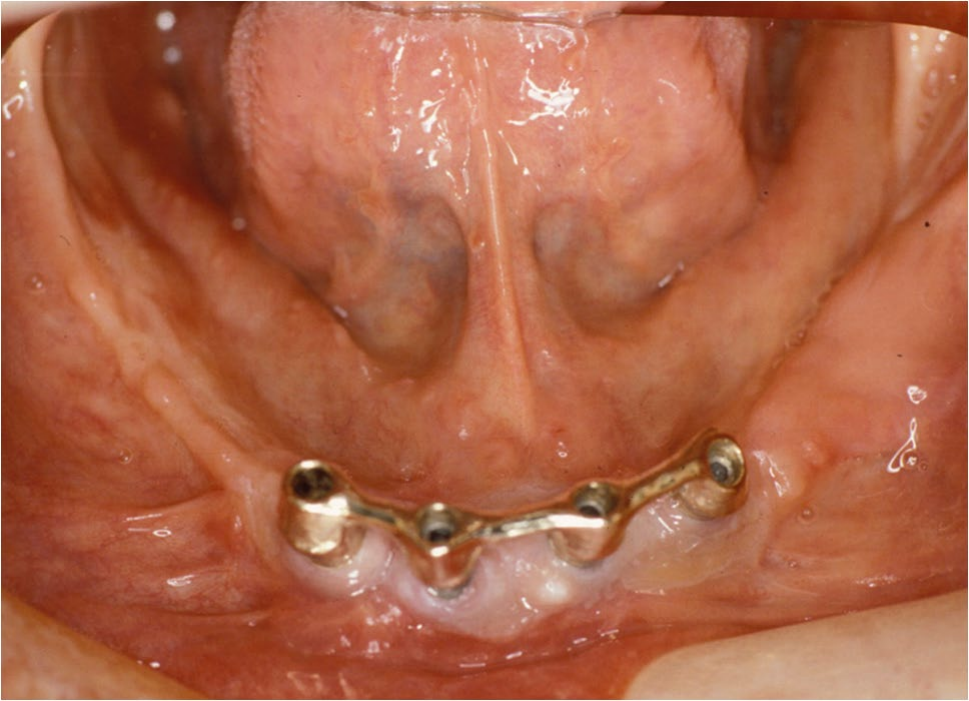
Prosthetic rehabilitation with overdenture 7 months after implant placement: intraoral view of the mandible; see soft tissues healing after skin graft harvested from the antero-lateral thigh

**Fig. 13:**
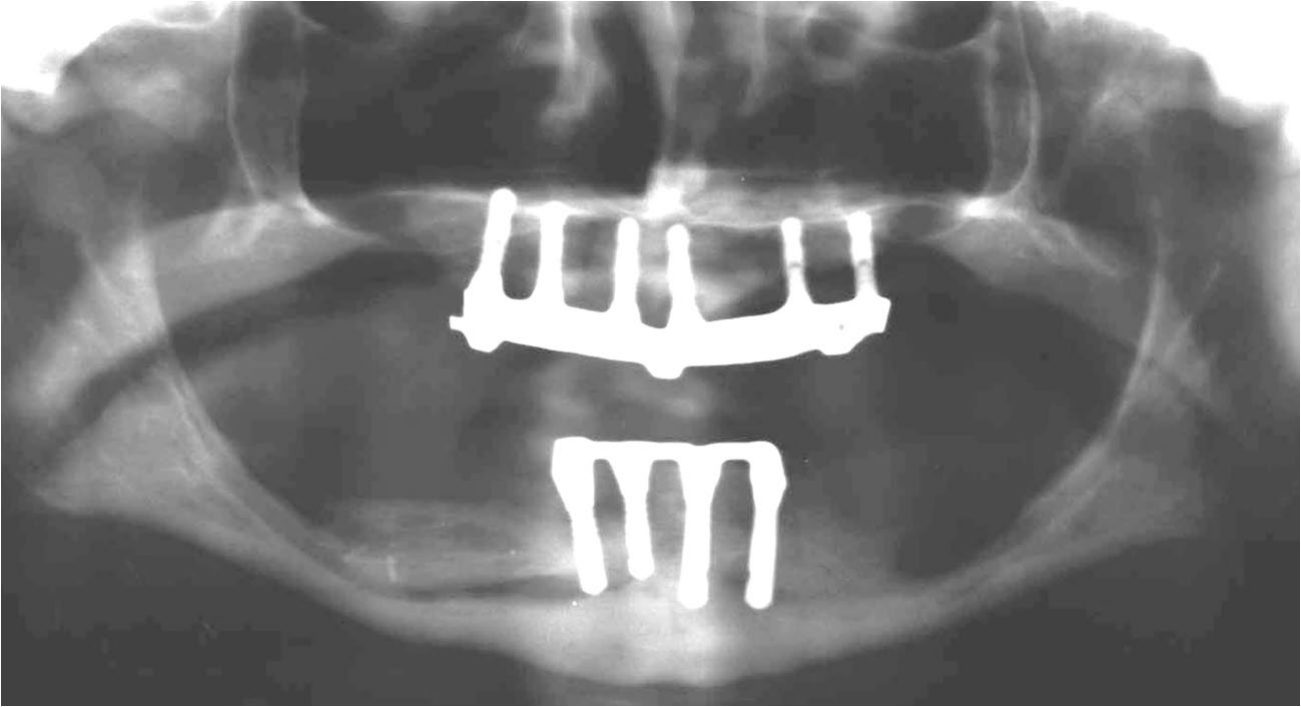
Prosthetic rehabilitation with overdenture 7 months after implant placement. X-ray panoramic radiograph taken after overdenture placement

**Fig. 14:**
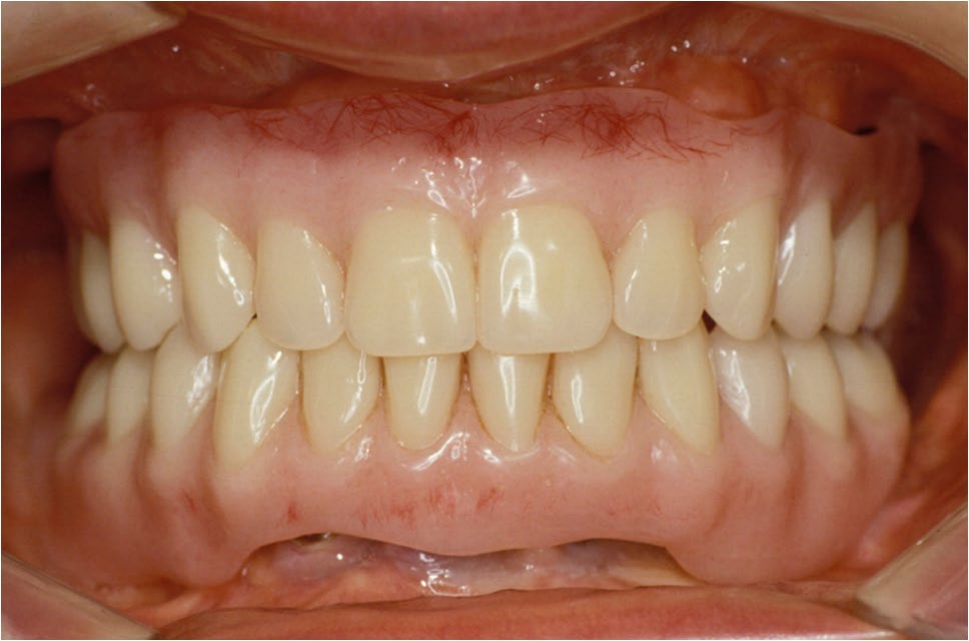
Prosthetic rehabilitation with overdenture 7 months after implant placement. Frontal view of prosthesis

**Fig. 15:**
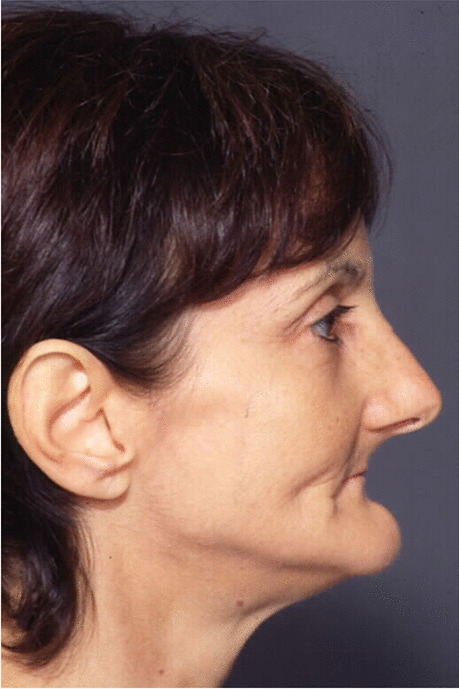
Follow-up evaluation 8 years after surgery. Lateral view of the patient at presurgical time

**Fig. 16:**
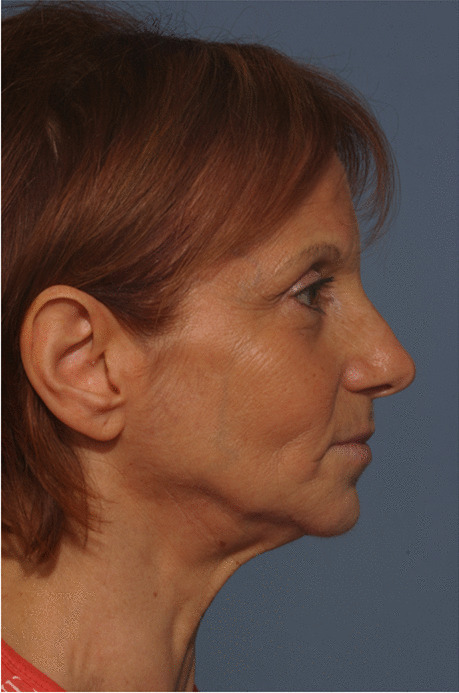
Follow-up evaluation 8 years after surgery. Lateral view of the patient at postsurgical time

**Fig. 17:**
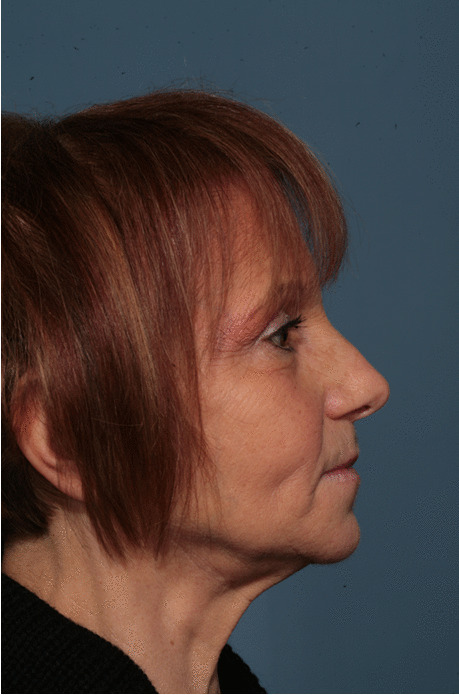
Follow-up evaluation 8 years after surgery. Lateral view of the patient at 8-year follow-up

**Fig. 18:**
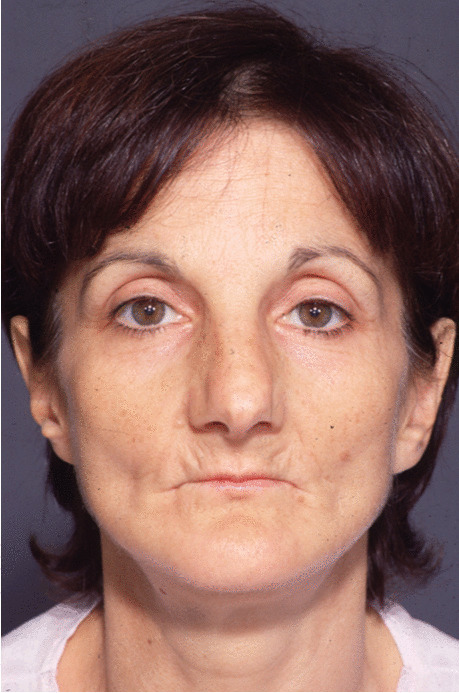
Follow-up evaluation 8 years after surgery. Frontal view of the patient at presurgical time

**Fig. 19:**
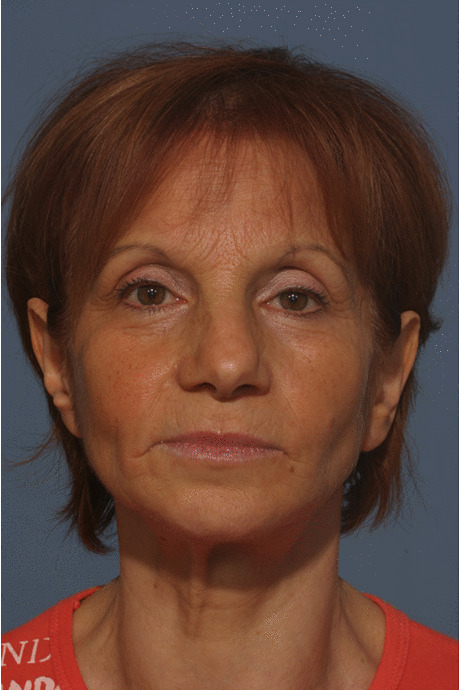
Follow-up evaluation 8 years after surgery. Frontal view of the patient at postsurgical time

**Fig. 20:**
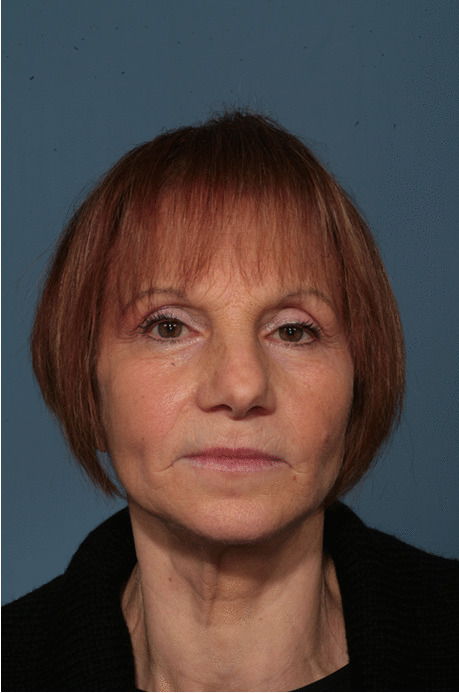
Follow-up evaluation 8 years after surgery. Frontal view of the patient at 8-year follow-up. See stability of aesthetic results, correction of pseudo-prognathism, and midface volume restoration

**Fig. 21:**
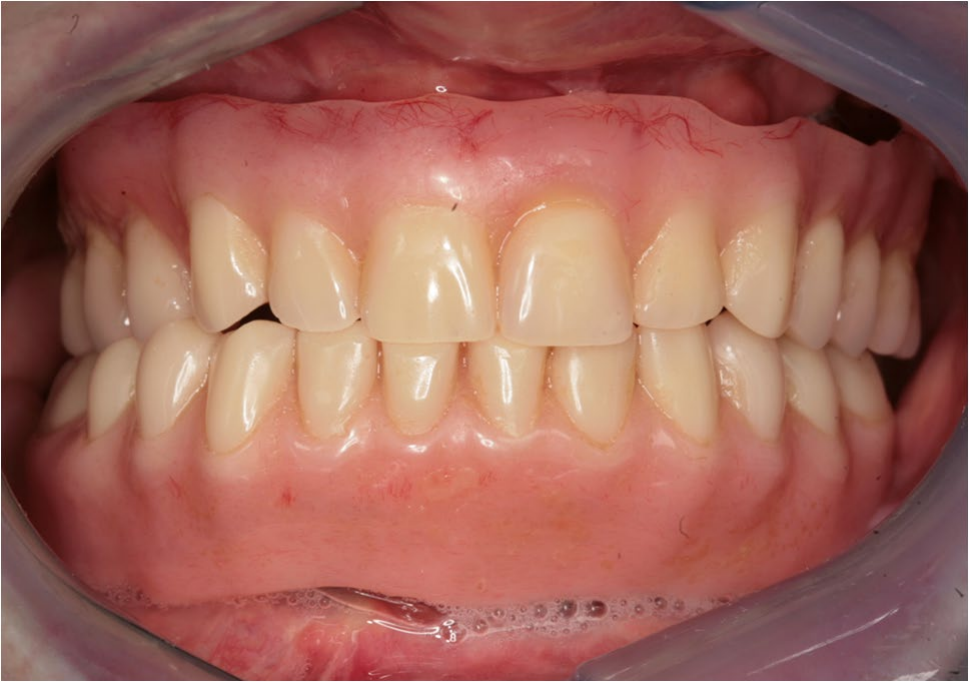
Follow-up evaluation 8 years after surgery. Frontal view with prosthesis

**Fig. 22:**
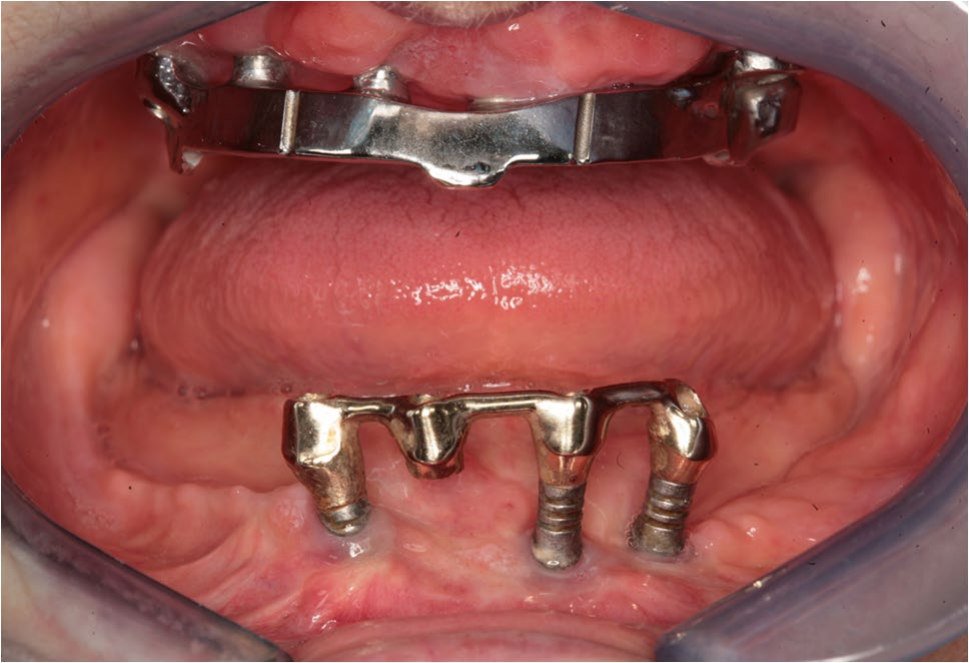
Follow-up evaluation 8 years after surgery. Frontal view after prosthesis removal. Exposed implant surface with peri-implantitis can be appreciated in 3.1, 3.3, and 4.3 sites; loss of implant can be seen at 4.1 site

**Fig. 23:**
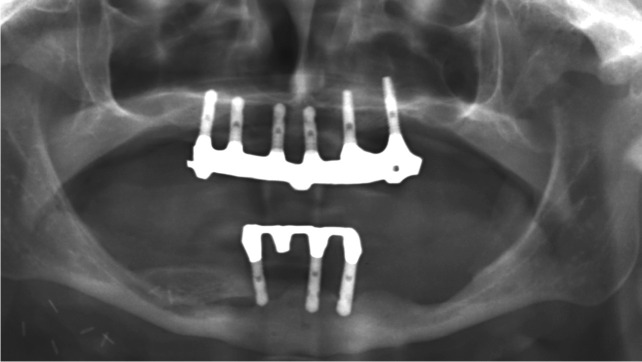
Follow-up evaluation 8 years after surgery. X-ray panoramic radiograph after prosthesis removal. Loss of implant can be seen at 4.1 site

Implant-prosthetic planning took place in two steps. Implants were uncovered 5–6 months after implantation: before uncovering, a panoramic radiograph was taken to assess the bone conditions. None of the implants was immediately loaded, and in all cases healing abutments were placed to allow complete soft tissues healing before the prosthetic phase. Depending on patient needs, bar-retained prostheses or screw-retained prostheses were employed. After surgery, all patients were prosthetically rehabilitated with overdentures or full-arch rehabilitation. All patients were called for one check-up soon after prosthetic rehabilitation. Thereafter, they were addressed to personal private dentists for routinely care and supportive periodontal therapy (SPT). A clinical and radiographic follow-up examination was also scheduled, to evaluate oral hygiene habits, soft tissues conditions, implant survival, and implant success (Figs. 1, 2). Figures 3, 4, 5, 6, 7, 8, 9, 10, 11, 12, 13, 14, 15, 16, 17, 18, 19, 20, 21, 22, and 23 show a clinical case of an upper full-arch prosthetic rehabilitation.

### Outcomes of the study: oral hygiene evaluation

At the follow-up examinations, a questionnaire was presented to assess patients’ ability on oral hygiene and to verify if they were involved in a correct supportive periodontal therapy (TPS). Each patient filled out the questionnaire independently, in the presence of a research assistant who answered eventual questions and offered explanations. The questions are reported as follows:

1. Are you inserted in a supportive periodontal therapy program and followed by a dental practice except to follow-up?
2. Do you regularly undergo oral hygiene sessions? If yes, how many times in a year?
3. Do you have any difficulties during home care oral hygiene?
4. Which instruments do you use for oral hygiene?

To also evaluate the access and capability in oral hygiene procedures at implant site (yes/no), patients were asked to show to brush the implant’s surfaces by means of oral hygiene instruments [22].

### Outcomes of the study: soft tissues conditions

At each follow-up session, the implant-supported prostheses were removed, and a clinical assessment was carried out. The peri-implant soft tissues conditions were collected by a single physician, using a standardized method with a calibrated plastic probe [4]. Four periodontal parameters were examined [4] and measured at mesial, vestibular, distal, and palatal/lingual side of the implant (Figs. 1 and 2): visible plaque index (VPI), bleeding on probing (BoP), probing pocket depth (PPD), and marginal recession (REC). Peri-implant infections with suppuration (SUPP) were also recorded.

### Outcomes of the study: implant survival and implant success

Implant survival (at implant level, CSR-Impl; at patient level, CSR-Pt) was defined as absence of implant failure, registered with presence of one of the following clinical conditions: implant-related pain on function; implant mobility; peri-implant radiolucency > 1/2 length of the implant; and/or implant removal caused by failure of osseointegration/peri-implantitis [30].

Prosthetic survival (CSR-Prosth) was evaluated as the presence of an intact and functional prosthetic rehabilitation.

Implant success (at implant level and patient level) was evaluated in case of no failure, absence of pain at implant site, and absence of peri-implantitis. Peri-implantitis was diagnosed when an implant had simultaneously one surface with positive BoP or pus on probing, and presence of radiographically detectable bone loss greater than 1.5 mm, plus < 0.2 mm per year of loading after the first year of service [31].

Peri-implant bone-level changes were recorded comparing panoramic radiographs taken at the time of prosthetic loading and at the time of follow-up examinations. Bone-level changes were evaluated at mesial and distal side of each implant, using a software program (Rasband, W.S., ImageJ, U.S. National Institutes of Health, Bethesda, Maryland, USA) measuring tool in conjunction with a magnification tool [3, 4]. The measurements were recorded to the nearest 0.5 mm; the distance between the implant shoulder and the first bone to implant contact point (F-BIC) was assessed [3, 4], and marginal bone loss (BL) was determined as the difference between the marginal bone level (F-BIC) at the follow-up time point and the marginal bone level at loading time.

### Data management and statistical analysis

For data collection, a database including all patients evaluated in the study was created with Microsoft Excel. All data analyses were carried out using Stata v.13.0 for Macintosh (StataCorp, College Station, TX, USA). The normality assumptions for continuous data were assessed using the Shapiro–Wilk test; mean and standard deviation were reported for normally distributed data, median and interquartile range (iqr) otherwise. For categorical data, absolute frequencies, percentages, and 95% confidence intervals were reported. The association between categorical variables was tested with the *χ*^2^ test; if any of the expected values was less than 5, a Fisher exact test was performed. The comparison between the means of two different groups was performed using unpaired Student’s “*t*,” or Wilcoxon rank-sum test. The comparison of the means among more than two groups was done using one-way analysis of variance (ANOVA), or Kruskal–Wallis equality-of-populations rank test as appropriate. Significance level was set at 0.05. The study presents compliance with the STROBE checklist guidelines [32].

## Results

In total, 25 patients (12 male and 13 female), with a mean age of 45.4 (range 19–66) years, underwent FFF surgical interventions between 01/01/1993 and 31/12/2007. The FFF were used for respectively 14 maxillary and 11 mandibular reconstructions. Nine patients were treated for severe atrophy due to history of periodontitis, 11 for tumor resection (6 irradiated and 5 non-irradiated for cancer patients), three for self-reported ballistic trauma, one for cleft palate, and one for osteomyelitis, for a total of 140 dental implants (81 in the upper jaw and 59 in the lower jaw) placed in the fibular bone for oral rehabilitation of these patients. Mean age was 46.2 ± 13.9 (range 19–67) months at implant surgery, 46.52 ± 13.95 (range 19–67) months at implant loading, and 52.4 ± 14.89 (range 21–72) months at the follow-up appointment. All implants were submerged for a mean integration time of 4.32 ± 1.89 (range 2–9) months. Fourteen patients received full-arch fixed prostheses and 11 removable bar-supported overdentures. After a mean follow-up from implant loading of 5.9 ± 3.8 (range 2–15) years, there were no dropouts, and all 25 patients were revaluated. At the follow-up evaluation, an assessment of prosthetic restorations was made to determine if access for oral hygiene had been provided (yes/no): half of the prostheses (52%) were judged not suitable to allow proper accessibility for oral hygiene.

### Patient-reported oral hygiene habits

Despite most of patients (68%) declared to give importance to oral hygiene recall appointments at the dental office, 40% never attended a dental visit, 52% had one oral hygiene session in a year, and only 8% had two oral hygiene sessions in a year. Only 28% of patients were properly followed by a dental hygienist: the lowest interest for oral health maintenance was shown by the group of irradiated tumor and ballistic trauma patients, for which no patient resulted involved in a SPT program; in the group of patients with history of periodontitis, only one out of the 9 patients demonstrated compliance.

A total of 60% of patients reported significant difficulties or even inability to perform oral hygiene procedures at home, and even two patients reported to not regularly perform oral hygiene at home. In total, 40% of patients reported to use only manual toothbrushing, while one patient declared to use only electric toothbrush. Sixty percent of patients referred to be compliant with interproximal oral hygiene: interdental brushing (83%), oral irrigator (5%), and interdental flossing with Superfloss™ (12%) were the most common additional techniques used. Moreover, patients with fixed prostheses, compared to removable bar-supported overdentures, referred greater difficulties in performing interproximal brushing. No patient reported to use any mouthwashes.

### Implant survival

None of the 25 prosthetic reconstructions failed, for a CSR-Prosth of 100%. Nine implants in seven patients resulted lost at evaluation time, for an overall CSR-Imp of 93.6% and CSR-pt of 72%.

Table 1 shows prevalence of implant survival (CSR-Imp) according to the following: sex, cause of atrophy, defect’s location, type of prosthesis, accessibility to implant neck for oral hygiene procedures at home, regularity of home-care oral hygiene procedures, self-reported difficulties in oral hygiene procedures, enrolment in SPT program, high VPI score (> 50%), interproximal oral hygiene measures.

**Table 1:** Prevalence of implant survival (*CSR-Imp*) according to study covariates; values are presented as *n* (%)

	Implant survival (%)	*p-*value
Sex		
M	91.3	0.72
F	95.8	
Cause of atrophy		
History of periodontitis	100	
Cancer irradiated for tumor resection	82.6	0.04*
Cancer non-irradiated for tumor resection	94.1	
Self-reported ballistic trauma	83.3	
Cleft palate	100	
Osteomyelitis	100	
Defect location		
Upper maxilla	95.1	0.06
Mandible	91.5	
Type of prosthesis		
Full-arch fixed	93.3	0.7
Removable overdenture	93.8	
Accessibility for oral hygiene		
No	90.9	0.08
Yes	96.8	
Regular homecare oral hygiene		
No	93.6	0.21
Yes	90.6	
Self-reported difficulty in performing oral hygiene procedures		
No	91.9	0.75
Yes	94.6	
SPT program		
No	89.3	0.09
Yes	94.7	
High VPI index (> 50%)		
No	92.4	0.07
Yes	94,1	
Interproximal oral hygiene		
No (only brushing)	84.6	0.03*
Yes	97.7	

*Statistically significant difference between groups

Regarding cause of atrophy, statistical differences were found between groups (*p* = 0.04). Assessing oral hygiene patients’ skills and possibilities to perform proper home oral hygiene, a significant greater implant survival was found for patients using interproximal oral health aids compared to the ones who did not perform interproximal hygiene measures (*p* = 0.03).

### Soft tissues conditions

Out of the 131 implants evaluated at the follow-up, presence of dental plaque and bleeding were respectively observed in 99 implants (VPI 75.6%) and 108 implants (BoP 82.4%), while suppuration was recorded in 30 implants (SUPP 22.9%). Mean PPD was 3.85 mm and mean REC was 2.11 mm.

Regarding cause of atrophy, high plaque and bleeding scores were found for all groups of patients, with no statistical differences among groups (*p* = 0.52 for VPI and *p* = 0.6 for BoP).

Regarding other groups of comparison, the highest plaque and bleeding scores were found in patients wearing prosthesis lacking accessibility for cleanability (respectively VPI of 73% vs 59%, *p* = 0.04; BoP of 85% vs 69%, *p* = 0.03), patients self-reporting difficulties in performing oral hygiene procedures (77.5% vs 50.9%, *p* = 0.001) and patients only brushing and not performing interproximal hygiene procedures (74.1% vs 61.2%, *p* = 0.05).

### Prevalence of peri-implantitis and implant success

Thirty-eight implants showed radiographical excessive bone loss and signs of inflammation; except for one patient, all other patients presented at least one implant with signs of peri-implantitis. Prevalence of peri-implantitis was 29% (38/131) at implant level and 96% (24/25) at patient level. Implant success was 70.9% (93/131) at implant level and 4% (1/25) at patient level.

Table 2 shows prevalence of peri-implantitis both at implant level and patient level, and prevalence of implant success, according to the abovementioned groups (same as for Table 1). Significant greater percentages of peri-implantitis at implant level (*p* < 0.05) were found: fixed prostheses, no adequate accessibility for oral hygiene procedures, self-reported difficulties in oral hygiene, patients not involved in a SPT program, high VPI score. Same outcomes were found for peri-implantitis at patient level (*p* < 0.05) for the last three variables. Accessibility for oral hygiene was the only variable found as significantly greater for implant success (*p* = 0.02).

**Table 2:** Prevalence of peri-implantitis at implant level and patient level, prevalence of implant success, according to study covariates; values are presented as *n* (%)

	Peri-implantitis implant level (%)	*p-*value	Peri-implantitis patient level (%)	*p-*value	Implant success (%)	*p-*value
Sex						
M	32.4	0.3	75	0.23	67.6	0.58
F	21.7		53.8		78.3	
Cause of atrophy						
History of periodontitis	31.4	0.46	88.8		68.6	
Cancer irradiated for tumor resection	41.7		57.1		58.3	
Cancer non-irradiated for tumor resection	20.4		50	0.3	79.6	0.08
Self-reported ballistic trauma	22.2		33.3		77.8	
Cleft palate	12.5		100		87.5	
Osteomyelitis	0		0		100	
Defect location						
Upper maxilla	28.7	0.95	78.6	0.07	71.3	0.3
Mandible	24.5		45.5		75.5	
Type of prosthesis						
Full-arch fixed	33.5	0.01*	71.4	0.48	66.5	0.057
Removable overdenture	18.4		54.5		81.6	
Accessibility for oral hygiene						
No	33.5	0.04*	69.2	0.94	66.5	0.02*
Yes	19.7		58.3		80.3	
Regular homecare oral hygiene						
No	24.1	0.06	60	0.09	75.9	0.63
Yes	21.1		47.1		78.9	
Self-reported difficulty in performing oral hygiene procedures						
No	5	0.001*	33.3	0.04*	95	0.09
Yes	39.1		81.3		60.9	
SPT program						
No	31.3	0.03*	70	0.02*	68.7	0.4
Yes	9.2		40		90.8	
High VPI index (> 50%)						
No	16.7	0.01*	28.6	0.001*	83.3	0.74
Yes	30.8		77.8		69.2	
Interproximal oral hygiene						
No (only brushing)	24	0.37	62.5	0.44	76	0.85
Yes	21.6		56.3		78.4	

*Statistically significant difference between groups

## Discussion

Studies in literature suggest the FFF as a reliable reconstructive surgical technique: implants placed in the reconstructed areas demonstrated proper integration with good long-term prognosis and high percentages of implant survival [18–20]. Investigations of the last decades [33] showed an overall implant survival between 82.4 and 100%, with follow-ups from 25 months to 20 years. The present 6-year retrospective study on 140 dental implants placed in FFF, used for the reconstruction of severely atrophic edentulous maxillae, showed an implant survival of 93.6% and a prosthetic survival of 100%. These findings are comparable to those published by several authors, who registered an overall implant survival of 93.5% on average, with a range of percentages between 83 and 97% from 1 to 5 years after placement [1, 21, 34–49], and a mean 5-year survival rate of 91% [50]. Lower values of 5-year implant survival (81% [51], 85.6% [52], and 87.2% [53]) were reported in studies which underlined recurrence of tumor, soft tissue proliferation, and infection as main factors involved in implant failure. On the other hand, higher percentages of 97.2% [33] and 98% [48] were reported after a mean follow-up of even 8 years after implant loading. To sum up, the average reported 10-year and 20-year implant survival were respectively 80% (range 78–83%) [39, 47] and 69% [1]. These heterogeneous findings in literature could be related to different patients’ characteristics of the studied samples.

First, the present study presented lower implant survival for dental implants installed in the irradiated area (82.6%), compared to those in not irradiated bone (94.1%). These outcomes seem to agree with the following: (i) studies [54] which assessed that radiotherapy significantly affects outcomes both at implant and patient levels; (ii) studies which found that hypo-vascularization following bone irradiation represents a contraindication for dental implant placement [53]; and (iii) studies which assessed that implant placement in the region of irradiated flap is significantly associated with implant failure [55]. Similarly, other studies showed a significant lower implant survival after 5 years in irradiated patients (83.5%) compared to non-irradiated ones (94.2%) [33]. Nevertheless, some authors [56, 57] reported that timing of implant placement, together with proper and meticulous management, may provide better clinical outcomes even in patients with irradiated bone, and this issue is still considered widely debated in literature [36, 58].

Regarding arch, implants placed in the maxilla presented a greater implant survival compared to those in the mandible (respectively of 95.1% vs 91.5%). The location of dental implant placement on implant failure was evaluated in literature with conflicting results: some authors [59] reported more failures in the maxilla, while others [60] did not find any differences among arches.

As a recent relevant issue, high prevalence of peri-implant diseases is coherent with an increasing employment of dental implants. In agreement with other authors [36, 38], the present study demonstrated that the most common complication associated with implant placement and the most common cause of implant loss is peri-implantitis. After an average follow-up of 6 years after loading, peri-implantitis prevalence was 29% at implant level, a percentage slightly greater compared to outcomes reported in literature using the same criteria for implants placed in the native bone, depending on periodontal disease history and implant design [61, 62]. Moreover, a recent meta-analysis [63] reported lower peri-implantitis percentages: moderate/severe peri-implantitis was observed in 21.7% of the implants. Due to significant heterogeneity in case definitions, peri-implantitis prevalence greatly depends upon main criteria used in each study [64]: an overall prevalence between 1 and 45% is overall reported. At the patient level, even considering this aspect, percentage obtained in the present study (96%) was greater than the weighted mean prevalence of 18% and 22%, respectively, declared in recent meta-analyses [63, 65] and clinical studies [66, 67]. However, it has to be underlined that, as in this study each patient presented extensive rehabilitation with multiple implants, peri-implantitis analysis at the patient level considered the entire mouth.

Even if oral hygiene habits of patients with implants in FFF are rarely reported in the literature, the outcomes of this study are consistent with well-known findings declaring that poor oral hygiene practices and compliance by patients are significantly associated with peri-implant disease [22, 24, 68]. Moreover, despite most of the patients (68%) affirmed to give enough importance to oral hygiene, 40% of them reported not to attend at all any dental offices, plus a great percentage resulted not enrolled in a SPT program (72%), and 60% of patients reported significant discomfort and difficulties, or even inability, on performing homecare oral hygiene measures around implant. Plus, a relevant number of patients (40%) did not perform interproximal oral hygiene. Considering VPI score of ≤ 50% as the threshold for “good” oral hygiene conditions, the overall high plaque levels exhibited by patients of this study suggest that a lower patient’s ability in performing adequate plaque control and lack of SPT could be strongly associated with peri-implantitis. In this proposal, the efficacy of patient-performed interdental cleaning methods for peri-implant health is not yet established, and usually is not explicitly described even in mucositis intervention studies. One study about performed oral hygiene around full-arch implant-retained prostheses after instructions revealed generally poor conditions [22], but there are no similar studies regarding fixed partial dentures or single-crown prostheses. Moreover, this study showed that interproximal brushing is advisable and at the same time unlikely to be adequate in preventing peri-implant disease: as subjects often performed more than one interproximal cleaning method, further research concerning the efficacy of different interproximal cleaning methods is warranted. When the type of prosthesis and potential impact of oral hygiene practices were evaluated, patients referred greater difficulties in performing interproximal brushing with fixed prostheses, which, in comparison with removable prosthesis, presented similar implant survival but lower implant success, due to higher prevalence of peri-implantitis. Implant-supported fixed prostheses indeed require longer abutments and long-term maintenance, difficult to keep clean by the patient [69].

To sum up, it can be underlined that accessibility for oral hygiene measures resulted in the greatest impact on implant survival, implant success, and prevalence of peri-implantitis. Prostheses with better accessibility for oral hygiene presented higher implant survival (96.8% vs 90.9%) and implant success (80.3% vs 66.5%), lower prevalence of peri-implantitis (19.7% vs 33.5%), less plaque accumulation (59% vs 73%), and less BoP score (69% vs 85%). In accordance with these results, even if patients with overdentures generally report significantly lower overall satisfaction with chewing capacity and aesthetics and higher psychological discomfort [70], the authors suggest that fixed prostheses should only be conceived in patients with a high level of motivation, and be designed with a specific attempt to allow accessibility for oral hygiene measures.

In addition, the importance of optimal plaque control and adherence to a strict maintenance program were widely described in the literature as a gold standard for preventing biological complications in patients with history of periodontitis [71], which represents a critical issue in long-term maintenance of implants [72, 73]. In the present study, where patients’ oral hygiene habits and levels, together with their adherence to supportive maintenance protocols, all resulted extremely low, subjects affected by periodontitis presented a consistent prevalence of peri-implantitis both at implant and patient levels, but their implant survival was 100%. This result reflects that, in the absence of good oral practices, implant survival difference between patients with a history of periodontitis and general population may be negligible during the first 5 years of follow-up, but becomes more pronounced later [74, 75]. In this proposal, some authors [63] demonstrated that a 5-year follow-up is usually not sufficient to evaluate the differences of implant survival between groups. Studies with longer follow-ups are thus needed to evaluate the impact of history of periodontitis and lack of SPT on implant survival specifically in FFF patients.

Finally, limitations of this study regard a retrospective approach in a university setting and a small number of patients, despite the number of implants being substantial. On the other hand, that a long-term follow-up of 6 years in patients is not easy to evaluate in a regular recall program could be considered a valuable starting point for further assessment of clinical conditions of larger groups of patients undergoing this specific type of surgery.

## Conclusions

Findings of the present study underline the need by clinicians for a careful assessment, in reference to a specific implant therapy, of patient’s oral hygiene conditions, especially concerning prosthetic accessibility for oral hygiene procedures in patients who underwent FFF surgery for extreme atrophies (e.g., due to periodontitis or irradiation for cancer therapy).

Several systematic reviews and meta-analyses have confirmed that both homecare procedures and professional plaque control, in association with an efficient SPT recall program, may lead to decreased clinical signs of peri-implant inflammation, preventing the insurgence of peri-implant infections even in these patients. Poor patients’ oral hygiene habits and levels prior to implant rehabilitations, if not improved after implant therapy, may therefore have a consistent impact not only on implant failure, but also on peri-implant disease onset.

## Data Availability

The data presented in this study are available from the corresponding authors upon reasonable request.
